# Clinical benefits of FilmArray meningitis-encephalitis PCR assay in partially-treated bacterial meningitis in Israel

**DOI:** 10.1186/s12879-019-4348-x

**Published:** 2019-08-13

**Authors:** Yair Mina, Vered Schechner, Michal Savion, Dafna Yahav, Efraim Bilavsky, Nadav Sorek, Haim Ben-Zvi, Amos Adler

**Affiliations:** 1 Sackler Faculty of Medicine, Tel-Aviv University, Tel-Aviv, Israel; 2 Neurological Institute, Tel-Aviv Sourasky Medical Center, 6th Weizman street, Tel-Aviv, Israel; 3 Division of Epidemiology and Preventive Medicine, Tel-Aviv Sourasky Medical Center, Tel-Aviv, Israel; 4 Tel-Aviv District, Israel Ministry of Health, Tel-Aviv, Israel; 5 Infectious Diseases Unit, Rabin Medical Center, Beilinson Hospital, Petah Tikva, Israel; 6 Department of Pediatrics C, Schneider Children’s Medical Center, Petah Tikva, Israel; 7 Microbiology Laboratory, Rabin Medical Center, Beilinson Hospital, Petah Tikva, Israel; 8 Microbiology Laboratory, Tel-Aviv Sourasky Medical Center, Tel-Aviv, Israel

**Keywords:** Meningitis, Bacteriology, Polymerase chain reaction

## Abstract

**Background:**

Management of partially-treated, community-acquired bacterial meningitis (PCBM) is commonly compromised by lack of microbiological diagnosis. We aimed to analyze the impact of FilmArray Meningitis-Encephalitis (FA-ME) PCR on the management of PCBM.

**Methods:**

Comparison of treatment variables of PCBM cases between two periods, before (6.5 years, control group) and after (2 years, study group) the application of FA-ME PCR assay.

**Results:**

The total duration of antimicrobial treatment in the study group (*n* = 8) was significantly shorter than the control group (*n* = 23) (9.5 ± 3.7 days vs. 15.2 ± 5 days, *p* = 0.007). The percentage of narrow-spectrum regimens was significantly higher in the study group (78 ± 11% vs. 40 ± 9%, *p* = 0.03). There was a significant difference in implementation of antimicrobial chemoprophylaxis for close contacts (4/8 (50%) vs. 1/23 (4%), *p* = 0.01).

**Conclusions:**

The use of FA-ME PCR provides significant benefits in the management of PCBM by shortening duration of antibiotic treatment, increasing the use of narrow-spectrum regimens, and allowing proper administration of antimicrobial chemoprophylaxis.

**Trial registration:**

The study was approved and retrospectively registered by the Tel-Aviv Sourasky Medical Center (0378–17-TLV, 10/17/2017) and Rabin Medical Center (0270–18-RMC, 11/11/2018) Ethics committees and conforms to recognized standards.

## Background

Bacterial meningitis causes significant morbidity and mortality [1], and though its incidence has decreased thanks to development of vaccines [2], it was still as high as 1.38 per 100,000 per year in the United States in 2006–2007 [3] and 1.44 per 100,000 per year in England [4], with no significant change in case fatality rate compared to 1998–1999 [3]. Etiology of community-acquired bacterial meningitis beyond the neonatal period is limited to a small number of pathogens including *Listeria monocytogenes*, *Haemophilus influenzae*, *Neisseria meningitidis* and *Streptococcus pneumoniae* [3]. In Israel, conjugated pneumococcal vaccines did not significantly change the rate of pneumococcal meningitis in children [5].

Management of bacterial meningitis differs significantly according to the pathogen [6], and these differences in management carry clinical, epidemiological and financial significance. For example, pneumococcal infection requires 14 days of antibiotic treatment and adjunctive steroid treatment, but no antimicrobial chemoprophylaxis for close contacts, while a meningococcal infection requires only 5–7 days of antibiotic treatment without adjunctive steroid treatment, but necessitates chemoprophylaxis treatment.

While cerebrospinal fluid (CSF) cultures are the gold standard in diagnosis of bacterial meningitis [7], these tests are less than optimal due to delayed turn-around time and relatively low recovery rate [8]. This is mainly due to the increasingly common practice of administrating antimicrobial treatment prior to lumbar puncture (LP), which has been shown to substantially and rapidly cause sterilization of CSF [9–11]. Hence, other diagnostic methods became necessary [12].

Several studies have demonstrated the beneficial effect of PCR tests for diagnosis of bacterial meningitis compared to CSF culture [13–17]. However, most of these studies were using various in-house PCR assays that are not widely available.

FilmArray Meningitis-Encephalitis (FA-ME) PCR assay is the first commercial, random-access syndromic-bases assay for diagnosis of meningitis and has shown very high sensitivity and specificity in identifying pathogens of community-acquired bacterial meningitis [18]. Thus, it can provide a rapid and reliable diagnosis of the microbial etiology in cases of culture-negative bacterial meningitis. The goals of our study were a) to study the frequency of partially-treated, community-acquired bacterial meningitis (PCBM) in our center, b) to study the microbial etiology of PCBM in our center and c) to study whether the application of FA-ME assay has led to changes in management of PCBM.

## Methods

### Setup

The Tel-Aviv Sourasky Medical Center (TASMC) is a tertiary care center, with 1500 inpatient beds of both pediatric and adult patients, and a stable occupancy of 100% along the year. In adults, CSF is collected by a lumber puncture only following head CT in the vast majority of cases and thus already after antimicrobials have been administered, whereas in children this policy is usually not practiced. The treatment of bacterial meningitis is generally based on international guidelines [7]. In adults, the use of empirical vancomycin was not generally recommended due to the very low rate of ceftriaxone-resistant *S. pneumoniae* in Israel [19] until the beginning of 2018. After few cases of ceftriaxone-resistant *S. pneumoniae* infections were diagnosed in TASMC (data not shown), vancomycin was also administered as empirical treatment for suspected *S. pneumoniae* meningitis.

In addition, the study included an observational-only part (see below) that was conducted at Rabin Medical Center (RMC). The RMC is also a tertiary care center that includes the largest pediatric hospital in Israel with 258 beds.

### Study design

This was a retrospective cohort study. Cases of PCBM were compared between two time periods: before (six and a half years) and after (two years) the introduction of the FA-ME PCR assay in our Clinical Microbiological Laboratory (on June 2016). The following criteria were used in order to define partially-treated, community-acquired bacterial meningitis (PCBM). Inclusion criteria were: 1) **Laboratory**: i) CSF with white blood cells (WBC) count of > 100 cells/μl. In addition we also evaluated all cases where CSF WBC count was not reported due to bloody tap; ii) negative CSF and blood cultures; iii) positive FA-ME PCR for bacterial pathogens (second period only). 2) **Clinical-demographic**: i) age > 3 months; ii) antimicrobial treatment prior to LP; iii) clinical diagnosis and treatment as bacterial meningitis. Exclusion criteria were: i) alternative diagnosis other than bacterial meningitis (infectious or non-infectious); ii) neurosurgical procedures/head trauma within 2 years prior to diagnosis. The second exclusion criterion was added since in these cases the variety of potential bacterial pathogens is larger than those included in the FA-ME PCR test (e.g., *Staphylococcus* spp.).

In addition to the main study in TASMC, we extended our study to include patients from RMC with PCBM that had a positive FA-ME PCR for bacterial pathogens. This part was observational only and intended to provide external validation for the conclusions drawn from our local study.

### Microbiological methods

The BioFire FilmArray (FA; Idaho Technology, Inc., Salt Lake City, Utah) meningitis/encephalitis (ME) assay is the first FDA-approved PCR-array test that can simultaneously detect multiple pathogens in a single CSF specimen. The ME assay identifies 6 bacterial pathogens, including *Escherichia coli* k1, *H. influenzae*, *L. monocytogenes*, *N. meningitidis*, *Streptococcus agalactiae* and *S. pneumoniae*, and also 7 viral pathogens and 1 fungal pathogen [3]. The test has been used in TASMC since June 2016 for any case of suspected PCBM based on the discretion of the laboratory director. In general, the main criterion to use this test was when full course of antimicrobial therapy for bacterial meningitis was deemed necessary. In RMC, the test has been used since July 2017.

Bacterial cultures were done according to the American Society of Microbiology guidelines [20]. Bacterial identification was done using VITEK2® or VITEK-MS® systems; blood cultures were processed using BACT/ALERT® 3D® or VIRTUO® systems (bioMerieux, Marcy l’Etoile, France).

### Data collection and analysis

Data were collected via chart review using electronic medical records. Data collected included patient characteristics, laboratory results and clinical parameters.

For the study group, we included all patients with a positive FA-ME PCR test result for bacterial pathogens since June 2016, who also fulfilled the other study criteria.

For the control group, the selection process was conducted as follows: a) Identification of all patients who underwent a LP in our hospital from January 2010 to May 2016; b) Review medical files of cases in which there was an abnormal CSF with WBC count > 100 cells/μl or a non-informative WBC-count due to technical difficulties; c) Exclusion of all patients from neonatal unit or neurosurgical department, and excluding cases with positive CSF or blood cultures; d) Cases were reviewed to identify patients who fulfilled the clinical inclusion criteria, including diagnosis and treatment as PCBM and documentation of antibiotics given prior to the LP.

After the identification of eligible cases, data were extracted from the medical files for the following variables:

1. Baseline patient information: age, gender, chronic diseases, any neurosurgical history, and date of admission.
2. Laboratory results: FA-ME PCR result (study group only), blood tests [glucose, WBC count, C-reactive protein (CRP), blood culture], CSF parameters (WBC count, proportion of polymorphonuclear cells, red blood cells count, protein, glucose, Gram stain, culture and any other microbiological or virological tests if performed), CSF:blood glucose ratio.
3. Clinical parameters: diagnosis at the end of hospitalization, length of stay (LOS), administration of steroids, admission and duration of ICU stay, in-hospital mortality, functional outcome (measured by modified Rankin Scale), and administration of antimicrobial chemoprophylaxis for close contacts, both in-hospital and in the community. The latter information was obtained from the District Public Health office.
4. Antimicrobial-related parameters (type and duration): the antimicrobial treatment regimen was divided into three periods: a) treatment prior to the diagnosis of PCBM (i.e., prior to performance of LP), including pre-admission therapy; b) initial treatment for bacterial meningitis (defined as the empiric treatment given for bacterial meningitis); c) final antimicrobial treatment (defined as the last regimen given to the patient after the change from the empiric treatment). The total duration of PCBM treatment was defined as the combined duration of the empiric and final antimicrobial regimens. Antimicrobial regimens were categorized as guideline-adherent if it followed the guidelines for the treatment of bacterial meningitis [7]. In the study group, the final regimen was categorized as guideline-adherent if it was changed according to the pathogen identified by the ME PCR assay. Additional parameters included the duration of narrow spectrum antibiotic treatment (defined as the use of a single antimicrobial agent other than carbapenems).

The primary outcome measure was defined as the total duration of antimicrobial treatment.

The effects on continuous variables were studied by t-test. The effect on the categorical variables was tested by chi-square test. All analyses were performed by IBM SPSS® Statistics version 25.

The study was approved by the TASMC and RMC Ethics committees and conforms to recognized standards.

## Results

### Frequency and microbial etiology of PCBM in TASMC

Between June 2016 and June 2018, we identified in the study group 8 patients with PCBM and a positive FA-ME PCR result that met all the study criteria, including 4 cases of *N. meningitidis*, 2 cases of *L. monocytogenes* and one case of *H. influenzae* and *S. pneumoniae,* each. One case occurred in 2016, 4 cases during 2017 and 3 cases during 2018. The microbial etiology is described in Table 1. For comparison, the number of culture-positive cases in the periods before and after the implementation of the FA-ME PCR were as follows: *S. pneumoniae*- 9 and 8 cases, *L. monocytogenes-*5 and 1 cases and *N. meningitidis-* 3 and 3 cases, respectively. Cases of PCBM with a negative FA-ME PCR results were not identified during the study period.

**Table 1:** Demographic, clinical and laboratory parameters of TASMC cases

	Study groupJune 2016 – June 2018	Control groupJanuary 2010 – May 2016	*p*-value
Number of cases	8	23	
Annual frequency (cases per year)	4	3.5	
Age (years)	40 ± 26	43 ± 20	0.73
Male, n (%)	4 (50)	7 (30)	0.57
Duration of antibiotic treatment prior to diagnosis (hours)	13 ± 18	77 ± 73	**0.02**
FilmArray ME PCR result	*N. meningitidis* (n = 4), *L. monocytogenes* (n = 2), *S. pneumoniae* (n = 1),*H. influenzae* (*n* = 1)	Not applicable	
Antimicrobials used in initial regimen			
Ceftriaxone	7 (88)	23 (100)	0.57
Ampicillin	3 (38)	8 (35)	0.89
Vancomycin	3 (38)	16 (70)	0.23
Acyclovir	2 (25)	7 (30)	0.77
Doxycycline	1 (13)	6 (26)	0.76
Other	0 (0)	4^a^ (17)	0.51
Antimicrobials used in final regimen			
Ceftriaxone	6 (75)	18 (78)	0.85
Ampicillin	2 (25)	5 (22)	0.85
Vancomycin	1 (13)	4 (17)	0.75
Acyclovir	0 (0)	1 (4)	0.55
Doxycycline	0 (0)	5 (22)	0.38
Other	0 (0)	2^b^ (9)	0.98
CSF-WBC (cells/μl)	3575 ± 3393	1186 ± 1429^c^	**0.01**
CSF-PMN-(%)	78 ± 24 ^d^	66 ± 32^c^	0.38
CSF-RBC (cells/μl)	26 ± 26	115 ± 201^c^	0.22
Glucose ratio (CSF:blood)	28 ± 26	44 ± 21^c^	0.11
CSF-Protein (mg/dL)	214 ± 121	183 ± 153	0.62
CSF-gram stain positive, n (%)	2 (25), 1-GP and 1-GN cocci	1 (4), GP cocci	0.31
Blood-WBC (10e3cells/ μl)	17 ± 8	16 ± 8	0.7
Blood-CRP (mg/L)	153 ± 107	80 ± 86	0.06
Chronic diseases	3 (38)	12 (52)	0.76
• Diabetes Mellitus	2 (25)	4 (17)	0.63
• Hypertension	2 (25)	4 (17)	0.63
• Vascular disease	2 (25)	1 (4)	0.16
(PVD/stroke/MI)			
• Other	1^e^ (13)	3^f^ (13)	0.97

*GP* gram positive, *GN* gram negative, *CSF* cerebrospinal fluid, *WBC* white blood cells, *PMN* polymorphonuclear, *RBC* red blood cells, *CRP* c-reactive protein, *PVD* peripheral vascular disease

Bold values are significant

^a^4 other antimicrobials used were clindamycin, oseltamivir, TMP-SMX, fluconazole

^b^2 other antimicrobials used were meropenem and moxifloxacin

^c^Only 22 cases were included in the analyses of these parameters due to missing data in one case with a traumatic lumbar puncture

^d^ Only 7 cases were included in the analyses of these parameters due to missing data in files.

^e^One patient with metastatic neuroendocrine tumor

^f^3 patients with HIV / Lymphoma / Multiple sclerosis

In order to identify patients in the control group, we reviewed medical files according to the process described above. We found a total of 21,714 patients who underwent LP from whom 7858 had an abnormal CSF with WBC count > 100 cells/μl or a non-informative WBC-count due to technical difficulties. After excluding patients from neonatal unit / neurosurgical department we were left with 4885 patients, of whom 4193 had a negative CSF culture. By reviewing the medical files of all these 4193 cases, we found 23 patients with a diagnosis of PCBM that fulfilled the study criteria including antibiotic treatment prior to LP and negative blood and CSF cultures. Four cases occurred in 2010, 2 cases in 2011, 4 cases in 2012, 6 cases in 2013, 4 cases in 2015 and 3 cases in 2016.

Considering the stable rate of hospitalized patients over the years we determined the combined average frequency of PCBM over 102 months as 3.65 cases per year (4 cases per year in the study group, and 3.5 cases per year in the control group).

### Clinical characteristics and outcome measures in TASMC

Table 1 summarizes the demographic, clinical and laboratory parameters of cases at TASMC. There were no significant differences between the groups in terms of demographic parameters, or the frequency of chronic diseases. Regarding laboratory parameters, there was a significant difference in the CSF white blood cell count (3575 ± 3393 cells/μl in the study group vs. 1186 ± 1429 cells/μl in the control group, *p* = 0.01) and a trend toward higher blood-CRP levels (153 ± 107 mg/L in the study group vs. 80 ± 86 mg/L in the control group, *p* = 0.06).

In terms of antimicrobial parameters, the mean duration of antibiotic treatment prior to the diagnosis of meningitis (i.e., results of CSF analysis) was shorter in the study group (13 ± 18 h in the study group vs. 77 ± 73 h in the control group, *p* = 0.02). There were only non-significant differences in the use of specific antimicrobial agents, although the final regimens were clearly more heterogenic in the control group, including 8 cases in which antibiotics other than ceftriaxone/ampicillin/vancomycin were used, as oppose to no such cases in the study group.

Table 2 shows comparison of the primary and secondary outcome measures in these cases. The total duration of antimicrobial treatment for PCBM was significantly shorter in the study group compared to the control group (9.5 ± 3.7 days vs. 15.2 ± 5.0 days, *p* = 0.007). The difference was mainly in the duration of the final antimicrobial regimen (8.6 ± 3.7 days vs. 12.8 ± 4.6 days, *p* = 0.03) whereas the duration of initial regimen was similar (3.5 ± 4.7 vs. 4.3 ± 3.4, *p* = 0.61).

**Table 2:** Comparison of primary and secondary outcome measures in TASMC groups

Variable	Cases (*n* = 8)	Control (*n* = 23)	*p*-value
Initial antimicrobial regimen duration (days)	3.5 ± 4.7	4.3 ± 3.4	0.52
Final antimicrobial regimen duration (days)	8.6 ± 3.7	12.8 ± 4.6	**0.03**
Total antimicrobial treatment duration (days)	9.5 ± 3.7	15.2 ± 3.7	**0.007**
Narrow spectrum antibiotic treatment duration (% from total duration)	78 ± 11	40 ± 9	**0.03**
Duration of guideline-adherent antimicrobial regimen (% from total duration)	93 ± 3	45 ± 9	**0.006**
Administration of steroids	3 (38)	9 (39)	0.94
Antimicrobial chemoprophylaxis for close contacts	4 (50)	1 (4)	**0.01**
In-hospital mortality	2 (25)	1 (4)	0.31
ICU admission duration (%)	21 ± 35	14 ± 26	0.5566
ICU admission	4 (50)	9 (39)	0.9
Length of stay (days)	10 ± 4	17 ± 9	0.065
Poor functional outcome (mRS ≥ 4)	2 (25)	3 (13)	0.82

Bold values are significant

The proportion of narrow-spectrum antimicrobial out of the total duration of treatment was higher in the study than in the control group (78 ± 11% vs. 40 ± 9, respectively, p = 0.03). In addition, the relative period of time in which a guideline-adherent treatment was given was significantly higher in the study group (93 ± 3% vs. 45 ± 9, *p* = 0.006).

When evaluating antimicrobial chemoprophylaxis for close contacts, we found documentation of in-hospital prophylaxis in a significantly higher percentage of cases in the study group (4 cases, 50%, in the study group vs. 1 case, 4%, in the control group, *p* = 0.01). These 4 cases in the study group required antimicrobial chemoprophylaxis for close contacts due to *N. meningitidis*. They were also notified to our local district health office and resulted in outpatient prophylaxis.

There was a trend toward a shorter length of stay in the study group (10 ± 4 days vs. 17 ± 9 days, *p* = 0.065). There were no significant differences in administration of steroids, patient mortality, ICU admission or the occurrence of poor functional outcome (mRS ≥ 4).

### Clinical characteristics and outcome measures in RMC

We included in this observational study, patients with PCBM from the RMC that were diagnosed by the FA-ME assay in order to evaluate whether the implementation was similar in regard to adaptation of antimicrobial treatment and chemoprophylaxis for close contacts. Between July 2017 and June 2018 there were 5 patients (including 3 children) with PCBM and a positive FA- ME PCR result that met all the study criteria. The microbial etiology was as follows: *N. meningitidis* (*n* = 4), *S. pneumoniae* (*n* = 1). The cases characteristics are summarized in Table 3. In all of these patients, after a short duration of empiric therapy (1.6 days in average) the regimen was changed to ceftriaxone based on the identification of the pathogen. The total antimicrobial treatment period was relatively short (8.6 days in average, with an average of only 7.3 days in the four cases of *N. meningitidis*) and was mostly narrow spectrum and guideline-adherent (averaging 79 and 90% respectively). Antimicrobial chemoprophylaxis for close contacts was administered in all 4 cases of *N. meningitidis*.

**Table 3:** Clinical, laboratory and outcome parameters of RMC cases

	RMC groupJuly 2017 – June 2018
Number of cases	5
Age (years)	17 ± 11
Male, n (%)	3 (60)
FilmArray ME PCR panel result	*N. meningitidis* (n = 4) *S. pneumoniae* (n = 1)
Antimicrobials used in initial regimen	
Ceftriaxone	5 (100)
Ampicillin	1 (20)
Vancomycin	3 (60)
Acyclovir	2 (40)
Doxycycline	1 (20)
Antimicrobials used in final regimen	
Ceftriaxone	5 (100)
CSF-WBC (cells/μl)	2626 ± 634
CSF-PMN-(%)	85 ± 4
CSF-RBC (cells/μl)	5 ± 5
Glucose ratio (CSF:blood)	56 ± 16
CSF-Protein (mg/dL)	228 ± 94
CSF-gram stain positive, n (%)	1 (20), GN cocci
Blood-WBC (10e3cells/ μl)	24 ± 4
Blood-CRP (mg/L)	183 ± 23
Chronic diseases	1 (20), mild developmental delay
Initial antimicrobial regimen duration (days)	1.6 ± 0.2
Final antimicrobial regimen duration (days)	7 ± 1.5
Total antimicrobial treatment duration (days)	8.6 ± 1.4
Narrow spectrum antibiotic treatment duration (% from total duration)	79 ± 5
Duration of guideline-adherent antimicrobial regimen (% from total duration)	90 ± 5
Administration of steroids	3 (60)
Antimicrobial chemoprophylaxis for close contacts	4 (80)
In-hospital mortality	0 (0)
ICU admission	4 (80)
Length of stay (days)	9.6 ± 2
Poor functional outcome (mRS ≥ 4)	0 (0)

*GP* gram positive, *CSF* cerebrospinal fluid, *WBC* white blood cells, *PMN* polymorphonuclear, *RBC* red blood cells, *CRP* c-reactive protein

## Discussion

Our study provides unique information about the clinical-epidemiological benefits of using the FA-ME PCR assay for the diagnosis PCBM. Previous studies have shown the analytical qualities of molecular tests for the diagnosis of bacterial meningitis [14–17]. The FA-ME PCR assay has shown high sensitivity and specificity [18, 21, 22] and cost effectiveness [23] in the diagnosis of central nervous system infections. However, these studies included also many viral meningitis-encephalitis cases and did not analyze the clinical benefits that were derived from the use of this test. Our study is the first to evaluate these benefits by comparing the clinical data of patients that were identified before and after the application of the test. We also aimed to evaluate the frequency of PCBM in the two periods. The identification of PCBM was straightforward following the introduction of the FA-ME assay but was extremely challenging in the control group, where cases had to be identified through a lengthy and complex procedure (described above). Interestingly, we found similar annual frequency in both groups, which supports the validity of the identification procedure.

Although our study group was small due to low incidence of bacterial meningitis, we were able to find significant differences between the groups that emphasize the advantages of the assay. We found significant benefits in the application of the assay in PCBM cases in our center, both for the individual patient and also for epidemiological purposes. This was manifested particularly in the shorter duration of antibiotic treatment and the ability to use mostly narrow-spectrum antibiotics. Although not reaching statistical significance (*p* = 0.065), the total length of stay was much shorter in the study group compared with the control (10 vs. 17 days respectively). Additional important benefit was the ability to identify and react to cases which required antimicrobial chemoprophylaxis for close contacts (e.g., *N. meningitidis*). Although the microbial etiology was unknown prior to the application of the FA-ME PCR assay (per study definition), it is likely that *N. meningitidis* infections had occurred and thus the opportunity for chemoprophylaxis was missed.

In addition to the analytical comparison, our data demonstrates the rationality that the use of molecular diagnostics can add to the management of these cases: immediately after a specific pathogen was identified, an effective guided therapy was administered for a well-defined period of time, as oppose to the somewhat chaotic management of patients in the control group, that in many cases received “non-conservative” antimicrobial regimens during some stages of their management. This was perhaps best demonstrated in the identification of patients with *Neisseria meningitidis* infection (4 out of 8 cases in the study group) in which the results enabled the choice of a narrow-spectrum antimicrobials for a shorter duration, and prompted the administration of antimicrobial chemoprophylaxis for close contacts, which was likely missed in some cases of culture-negative bacterial meningitis. In RMC, the introduction of the test allowed similar changes in management, both for the individual patient and for exposed personnel.

From demographic and clinical perspectives, there were no significant differences between the study and control groups in terms of age, chronic diseases or the need of ICU-admission. It is notable that there were some differences in the clinical and laboratory characteristics between the groups: CSF-WBC and blood-CRP level were lower in the control group, and these patients had a longer duration of antibiotic treatment prior to diagnosis. We think that these differences suggest that patients in the study group were in general suffering a more severe illness compared with the control group, which might have included cases of non-bacterial meningitis, causing a more insidious clinical presentation resulting in outpatient antibiotic treatment for other possible diagnosis (e.g. sinusitis). This emphasizes even more the advantage of using this test that can also serve to exclude bacterial meningitis and prevent the need for unnecessary prolonged courses of antibiotics. In addition to the clinical benefit, the FA-ME PCR assay may be cost-saving, but this has to be weighted with the high cost of the assay. Since the FA-ME PCR assay includes viral and fungal pathogens and therefore can be used for indication other than PCBM, it is difficult to estimate the ‘number needed-to-test’ for one confirmed case of PCBM.

The study has several limitations. First, the control group seems to be heterogeneous since these cases did not reach a definite diagnosis in the absence of a molecular diagnostic tool, and some of them might have been viral meningitis. Nevertheless, we believe that this heterogeneity reflects a real-life obstacle in identifying cases of PCBM, and thus emphasizes the importance of molecular diagnostics. Second, the retrospective nature of the study might potentially limit the reliability of the data collection, although most of the necessary clinical and epidemiological data was easily retrieved. Third, the low number of patients in the study group has limited our ability to reach statistical significance in some of the outcome measures (e.g., length of stay), yet significant differences were demonstrated in many parameters.

## Conclusions

In conclusion, our study demonstrates the significant clinical benefits that are offered by the use of rapid molecular diagnostic tool such as the FA-ME PCR in the management of PCBM.

## Data Availability

The datasets generated and/or analysed during the current study are not publicly available due institutional restrictions, but are available from the corresponding author on reasonable request.

## Abbreviations

CRP: c-reactive protein
CSF: Cerebrospinal fluid
FA-ME: FilmArray Meningitis-Encephalitis
GP: Gram positive
LP: Lumbar puncture
PCBM: Partially-treated, community-acquired bacterial meningitis
PMN: Polymorphonuclear
RBC: Red blood cells
RMC: Rabin Medical Center
TASMC: Tel-Aviv Sourasky Medical Center
WBC: White blood cells
